# Cancer Stem Cell-Like Side Population Cells in Clear Cell Renal Cell Carcinoma Cell Line 769P

**DOI:** 10.1371/journal.pone.0068293

**Published:** 2013-07-11

**Authors:** Bin Huang, Yi Jun Huang, Zhi Jun Yao, Xu Chen, Sheng Jie Guo, Xiao Peng Mao, Dao Hu Wang, Jun Xing Chen, Shao Peng Qiu

**Affiliations:** 1 Department of Urology, The First Affiliated Hospital, Sun Yat-Sen University, Guangzhou, China; 2 Department of Pharmacology, Zhongshan School of Medicine, Sun Yat-Sen University, Guangzhou, China; 3 Department of Urology, Sun Yat-Sen University Cancer Center, Guangzhou, China; Cedars Sinai Medical Center, United States of America

## Abstract

Although cancers are widely considered to be maintained by stem cells, the existence of stem cells in renal cell carcinoma (RCC) has seldom been reported, in part due to the lack of unique surface markers. We here identified cancer stem cell-like cells with side population (SP) phenotype in five human RCC cell lines. Flow cytometry analysis revealed that 769P, a human clear cell RCC cell line, contained the largest amount of SP cells as compared with other four cell lines. These 769P SP cells possessed characteristics of proliferation, self-renewal, and differentiation, as well as strong resistance to chemotherapy and radiotherapy that were possibly related to the ABCB1 transporter. *In vivo* experiments with serial tumor transplantation in mice also showed that 769P SP cells formed tumors in NOD/SCID mice. Taken together, these results indicate that 769P SP cells have the properties of cancer stem cells, which may play important roles in tumorigenesis and therapy-resistance of RCC.

## Introduction

Renal cancer is an important health problem, causing over 15,000 deaths in North America annually. Renal cancer with metastasis or at advanced stage in adults is resistant to conventional chemotherapeutic drugs [1]. Elucidating the genesis of this cancer will help the early diagnosis and treatment, thereby improving the prognosis.

Solid tumors are composed of diverse types of cells with different capacity of proliferation. Only a small population of these cells can form tumors in immunodeficient mice [2]. This observation has led to the concept of cancer stem cells (CSCs), so-called tumor-initiating cells or stem-like cancer cells [3], [4], [5], [6], which have been thought capable of proliferating, self-renewing, and differentiating into multiple lineages, thereby playing an essential role in both development and treatment of tumors [2], [3]. Although CSCs have been isolated from several types of human tumors, including hematologic cancers [7], ovarian cancer [8], prostate cancer [9], breast cancer [10], and brain tumors [11], the lack of CSC-specific cell surface antigen markers has bounded further investigation on this topic [12]. Side population (SP) is a flow cytometry (FCM) term to define cell clusters with strong ability to efflux DNA dye Hoechst 33342 via ABC transporters. Side population cells disappear upon treatment with either calcium channel blockers or inhibitors of ABC transporters, such as verapamil and rapamycin [13].This activity leads to the “side” (low fluorescence) phenotype of the population and is believed to be a fundamental self-protective function and thus a universal hallmark of stem cells [14], [15]. Since it was first introduced by Goodell et al. in 1996 [16], SP cells have been widely reported to be enriched in various cancerous tissues such as breast cancer [17], gastrointestinal system tumor [18], and small-cell lung cancer [19] and from cell lines such as nasopharyngeal carcinoma [20], hepatocellular carcinoma [21], and bladder cancer cell lines [22]. SP cells, with stemness potentials, can form xenograft tumors in animals and are resistant to chemotherapy and radiotherapy, contributing to tumor relapse [23].

RCC, the third most common cancer of the urinary tract, accounts for approximately 3% of all human malignancies. RCCs are classified as clear cell, papillary, chromophobe, collecting duct, and unclassified RCC, with clear cell RCC (CCRCC) as the most prevalent type. That accounts for 82% of RCCs. The treatment of metastatic CCRCC remains to be a major challenge for clinicians and causes approximately 35% of RCC-related mortality [24]. RCC cases have been increasing steadily for decades [25]. Furthermore, most patients already have either metastatic disease at the initial diagnosis or distant metastases after primary tumor resection [26]. The prognosis of RCC is poor partly due to the resistance of metastatic RCC to most current therapies, such as chemotherapy and radiotherapy. Targeted therapy against CSCs may bring new hope for improving prognosis of patients with RCC.

Although significant progress has been made in SP research, the role of SP cells in RCC remains to be fully determined [27], [28], [29], [30]. Addla et al. [29] have reported that both normal and malignant renal epithelial cells contained a proportion of SP cells which were enrich with some stem cell-like properties. More recently, Nishizawa et al. [30] have found that SP cells derived from RCC cells showed higher tumor-initiating ability than NSP cells. Therefore, we hypothesized that SP cells are an enriched fraction of cancer stem cells.

The present study was undertaken to identify the SP cells from established human RCC cell lines and to determine their characteristics and roles in tumorigenesis and treatment of RCC. Here, we isolated SP cells from 769P cells, a human CCRCC cell line, by Hoechst staining and flow cytometry. Our in vitro and in vivo experiments demonstrated that SP cells possessed the well-known CSC characteristics of proliferation, self-renewal, and differentiation, as well as strong resistance to chemotherapy and radiotherapy that were possibly related to the ABCB1 transporter. These findings may provide new insights for future CSC research and clinical anti-cancer therapy.

## Materials and Methods

### Cell Culture

Five human RCC cell lines 769P, 786-O, OS-RC-2, SN12C, and SKRC39 were used in this study. Cell lines 769P and 786-O were obtained from the American Type Culture Collection (ATCC, Manassas, VA, USA); OS-RC-2, SN12C, and SKRC39 were kindly gifted by Dr. Zhuowei Liu (Department of Urology, Sun Yat-sen University Cancer Center), who obtained these cell lines from the Type Culture Collection of Chinese Academy of Sciences (Shanghai, China) [31], [32], [33]. 769P, 786-O, OS-RC-2, and SKRC39 cells were cultured in RPMI-1640 (Gibco, Carlsbad, California, USA), whereas SN12C cells were cultured in Dulbeccos’s modified Eagle’s medium (DMEM, Gibco) at 37°C in a 5% CO_2_ atmosphere. Both media contained 10% fetal calf serum (FCS, Gibco), 1% (v/v) penicillin, and 100 µg/mL streptomycin.

### Side Population Analysis and Cell Sorting

Side population analysis and cell sorting were performed as described previously by Goodell et al. [16] with modification. Briefly, cells were trypsinized, suspended at 1 × 10^6^ cells/mL in pre-warmed RPMI-1640 containing 2% FBS and 10 mmol/L HEPES (Gibco), then incubated with 5 µg/mL Hoechst 33342 (Sigma, St. Louis, MO, USA) either alone or in combination with 50 µmol/L verapamil (Sigma), an ABC transporter inhibitor, in dark for 90 min in the 37°C water bath with intermittent mixing. At the end of staining, cells were spun down and resuspended in cold HBSS (Gibco) containing 2% FBS and 10 mmol/L HEPES. FCM analysis and cell sorting were then carried out directly on EPICS ALTRA Flow Cytosorter (Beckman Coulter, Fullerton, CA, USA). Hoechst 33342 was excited with 100 mW UV laser and was detected with 450 BP filter for blue fluorescence and 675 BP filter for red fluorescence. A 610-*nm dichroic mirror short*-*pass* (DMSP) filter was used to separate the emission wavelengths. A polygonal live gate in FS-HO blue plot was created to exclude debris and dead cells. SP cells and non-SP (NSP) cells were sorted for the following assays.

### Clone Formation and Long-term Differentiation Assays

Under the autoclone sorting mode, every 500 769P cells of SP or NSP phenotype were sorted directly into a 6-cm culture dish, and cultured with RPMI-1640 complete culture medium for 10 days. After most cell clones had expanded to more than 50 cells, they were washed twice with PBS, fixed in 75% methanol for 15 min, and stained with crystal violet for 15 min at room temperature. After incubation, dishes were rinsed and the number of clones that contained more than 50 cells was counted under a phase contrast microscope. The clone formation efficiency was the ratio of the number of clones to the number of seeded cells. Clone formation assays were repeated in triplicate.

The long-term differentiation assay was performed 10 days after cell sorting, according to the protocol of side population analysis, to determine the differentiation ability of SP and NSP cells.

### Detecting mRNA Expression of ABC Family Members in Sorted 769P SP Cells by RT-PCR

Total RNA was extracted from SP and NSP cells separately using Trizol reagent (Invitrogen, San Diego, CA). The expression of ABCB1, ABCC1, and ABCG2 was detected by using the PrimeScript^TM^RT-PCR Kit (Takara, Otsu, Japan) according to the manufacturer’s instructions. The primers (Table 1) were designed and synthesized by Invitrogen. GAPDH was used as an internal reference. The PCR conditions were denaturation at 94°C for 4 min followed by 40 cycles of annealing at 94°C for 45 s, 58°C for 30 s, and 72°C for 45 s, with elongation at 72°C for 8 min. The PCR products were analyzed by electrophoresis on 1.5% agarose for the mRNA expression of ABC family members.

**Table 1:** Sequences of primers used in RT-PCR.

Gene	Primer sequences	Productlength (bp)
ABCB1	Forward, 5′-TTTGGTGCCATGGCCGTGGG-3′Reverse, 5′-CGATGCCCAGGTGTGCTCGG-3′	400
ABCC1	Forward, 5′-ATGTCACGTGGAATACCAGC-3′Reverse, 5′-GAAGACTGAACTCCCTTCCT-3′	349
ABCG2	Forward, 5′-GGGTTCTCTTCTTCCTGACGACC-3′Reverse, 5′-TGGTTGTGAGATTGACCAACAGACC-3′	389
GAPDH	Forward, 5′-GAGTCAACGGATTTGGTCGT-3′Reverse, 5′-GACAAGCTTCCCGTTCTCAG-3′	185

### Detecting Protein Expression of ABC Family Members in Sorted 769P SP Cells by Western Blotting

Total proteins were extracted from SP and NSP cells separately and denatured in sodium dodecyl sulfate (SDS) sample buffer, then equally loaded onto 8% polyacrylamide gel. After electrophoresis, the proteins were transferred onto a polyvinylidene difluoride membrane. Blots were incubated with indicated primary antibodies overnight at 4°C, then incubated with horseradish peroxidase-conjugated secondary antibody, and were finally detected using enhanced chemiluminescence Western blotting detection reagents (GE healthcare, UK). The mouse ABCG2 (at 1∶1,000 dilution), ABCB1 (1∶1,000), and ABCC1 (1∶5,000) monoclonal antibodies from Abcam Inc. (Cambridge, UK) as well as anti-GAPDH (1∶2,000) from Santa Cruz Biotechnology (Santa Cruz, CA, USA) were used to determine relative protein levels.

### Radiation and Drug Sensitivity Assays

To determine the sensitivity of cells to radiation, freshly sorted SP and NSP cells were seeded in 6-well plates (500 cells/well), and were exposed to 5 Gy of X-ray (500 cGy/min, using a 12 cm × 6 cm irradiation field) with or without 30-minute pre-incubation with verapamil (50 µmol/L) the day after sorting. When most cell clones had more than 50 cells, they were stained with crystal violet to determine the number of survival cells.

To determine the sensitivity of cells to drugs, SP and NSP cells were seeded in 96-well plates (500 cells/well) and cultured with mitoxantrone (MTX, a topoisomerase II inhibitor antineoplastic agent, Sigma), 5-fluorouracil (5-FU, Sigma), or sunitinib (a tyrosine kinase inhibitor, Sutent, Pfizer, New York, USA) the following day in a concentration gradient, with or without 30-minute pre-incubation with 50 µmol/mL verapamil as a chemosensitizer to mitoxantrone. Untreated cells were used as control. Four parallel wells were set for each group. After 3 days, the absorbance of each well at a wavelength of 570 nm (*A*_570_) was measured. Cell survival rate was calculated using the formula: survival rate = (mean *A*_570_ of the test wells/mean *A*_570_ of the control wells) × 100%. Inhibition rate was calculated using the formula: inhibition rate = 100% - survival rate.

### Xenograft Tumor Formation Assay

Animal experiments were performed in strict accordance with the Guide for the Care and Use of Laboratory Animals of Sun Yat-sen University. The protocol was approved by the Committee on the Ethics of Animal Experiments of the First Affiliated Hospital of Sun Yat-sen University. A total of 54 5- to 7-week-old nonobese diabetic (NOD)/severe combined immunodeficient (SCID) female mice were obtained from the Experimental Animal Center of Sun Yat-sen University. The mice were divided into 6 groups, with 9 mice in each group. Indicated amount of freshly sorted SP and NSP cells were suspended in 200 µL PBS, separately, and inoculated subcutaneously into the axillary fossa of NOD/SCID mice immediately after the sorting. The mice were monitored twice per week for the formation of palpable tumors. At 6 weeks after inoculation, the mice were euthanized to assess tumor formation. Tumors were measured using a Vernier caliper, weighed, and photographed. A portion of every subcutaneous tumor was collected, fixed in 10% formaldehyde, and embedded in paraffin for pathological assessment after H&E staining. The other portion of every tumor was dissociated into single cell suspension, which was prepared as described previously [34] with minor modifications. Briefly, tumor tissue was manually dissociated into <0.5 mm fragments and all visible clumps were removed, then digested with 1 mg/mL collagenase type II (Sigma) and 1.2 mg/mL Dispase (Sigma) for 45 to 90 min at 37°C. Occasionally, 0.2 µg/mL trypsin (Invitrogen) was used for 10 min to ensure dissociation into single cells. Cells were filtered through consecutive 70-µm cell strainers to remove remaining clumps. Collected cells were suspended in PBS supplemented with 1% FCS. At least 100,000 harvested cells were stained for SP analysis. Twenty 5- to 6-week-old SDIC female mice were divided equally into 4 groups for serial transplantation assay. Every 5,000 cells dissociated from a xenograft tumor, which was formed with 2,000 SP cells, 20,000 SP cells, 20,000 NSP cells, or 200,000 NSP cells, were re-inoculated into a NOD/SCID mouse. Tumor formation assessment and pathological examination were performed 6 weeks later.

### Statistical Analysis

Data are expressed as the mean ± standard deviation (SD) from at least three independent experiments. Microsoft Office Excel 2007 and SPSS13.0 softwares were used for data processing. Statistical significance was determined with Student’s *t* test. A *P* value <0.05 was considered significant.

## Results

### Existence of SP Cells in RCC Cell Lines

**Figure 1:**
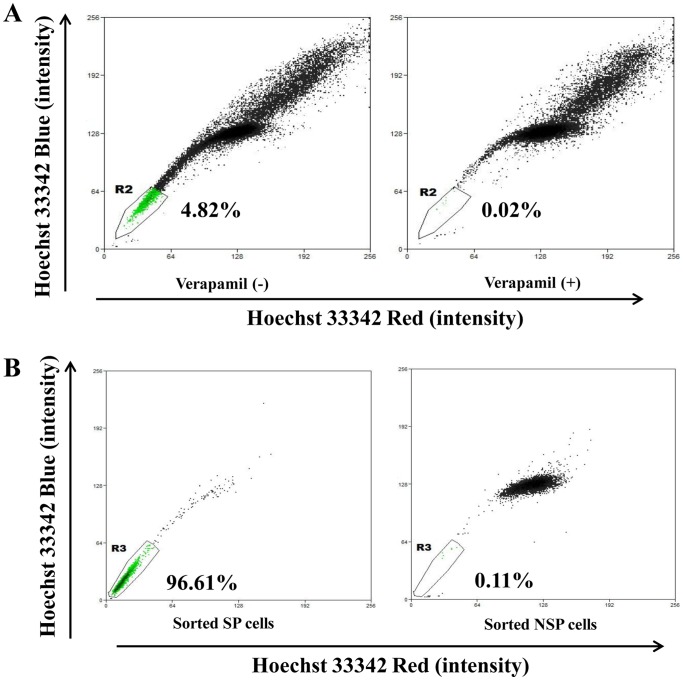
Side population (SP) cell sorting results and sorting purity in human renal cell cancer cell line 769P. **A**, SP sorting using Hoechst 33342. A polygonal live gate was created to exclude debris and dead cells. At least 50,000 cells were acquired to analyze the SP phenotype of every sample. The percentage of SP cells dropped when 769P cells were pre-incubated with verapamil to block the ATP transporter. **B**, the purity of freshly sorted 769P SP and non-SP (NSP) cells was 96.61% and 99.89% (1–0.11%).

Five RCC cell lines were analyzed for their SP phenotypes. The R2 gate shows that the percentage of SP cells, with dim Hoechst 33342 fluorescence, dropped from 4.82% among 769P cells without verapamil treatment to 0.02% among 769P cells with verapamil pre-incubation (Fig. 1A). Retesting of sorted cells demonstrated a purity of 96.61% for SP cells and 99.89% for NSP cells (Fig. 1B). For the other four RCC cell lines, the ratios of SP cells in 786-O and OS-RC-2 cells were 0.1% and 0.2%, which were too low for the following experiments; no SP cells were detected among SN12C and SKRC39 cells (Fig. S1). Therefore, SP and NSP cells were sorted from 769P cells for subsequent experiments.

### Clone Formation and Differentiation of Sorted 769P SP and NSP Cells

**Figure 2:**
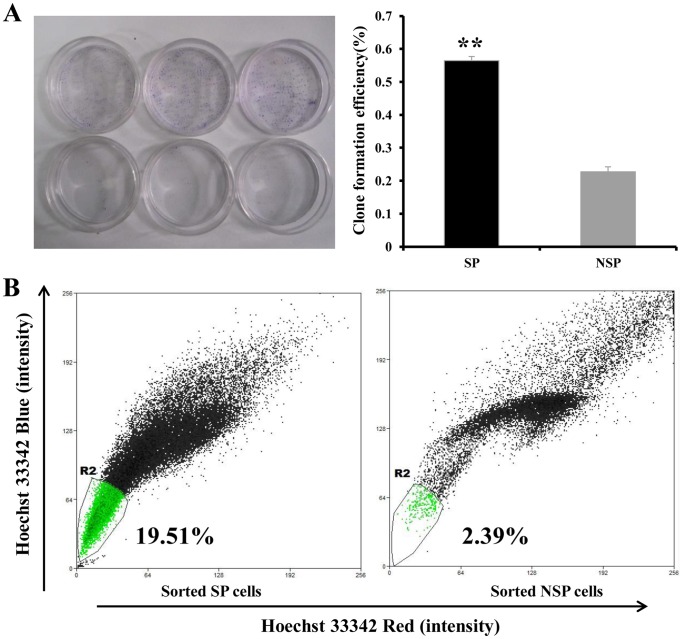
Clone formation and long-term culture differentiation of sorted 769P SP and NSP cells. **A**, clone formation assays reveal that the clone formation efficiency of freshly sorted SP cells was higher than that of NSP cells. **, *P*<0.01. **B**, at 10 days after sorting, the proportion of SP cells (indicated by the R2 gate) among sorted SP cells and sorted NSP cells were measured. The proportion of SP cells among sorted SP cells is only 19.51%, suggesting that most sorted SP cells are differentiated into NSP cells. The proportion of SP cells among sorted NSP cells is only 2.39%, suggesting that sorted NSP cells cannot differentiate into SP cells.

After 7 days of culture, most clones contained more than 50 cells. We counted the number of clones and found that the mean clone formation efficiency of SP cells was significantly higher than that of NSP cells [(56.4±1.3)% vs. (22.7±1.5)%, *P*<0.001; Fig. 2A).

After 10 days of culture, most sorted 769P SP cells differentiated into NSP cells, whereas only a small proportion of SP cells were detected among sorted 769P NSP cells (Fig. 2B), suggesting the ability of SP cells to self-renew and differentiate into NSP cells.

### Expression of ABC Family Members in Sorted 769P SP and NSP Cells

**Figure 3:**
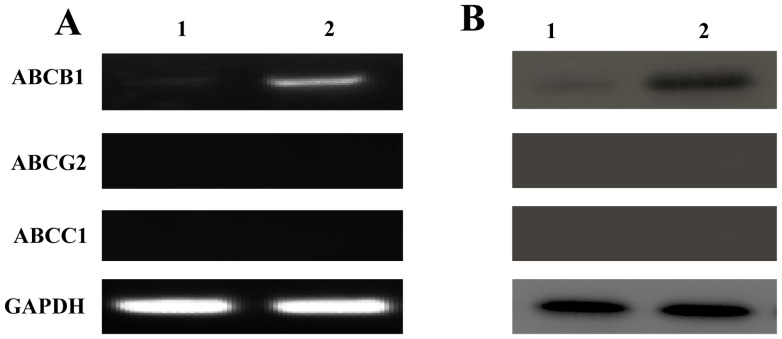
Expression of ABC family members in sorted 769P SP and NSP cells. **A**, reverse transcription-polymerase chain reaction. **B**, Western blotting. Lane 1, sorted NSP cells; lane 2, sorted SP cells. GAPDH was used as the internal control. Both experiments show that ABCB1 expression is stronger in SP than in NSP cells and that ABCG2 and ABCC1 are not expressed in SP and NSP cells.

The expression of ABCB1, ABCG2, and ABCC1 was detected by RT-PCR and Western blotting. Both experiments showed that ABCB1 was expressed at a high level in sorted SP cells but at a quite low level in sorted NSP cells, whereas ABCC1 and ABCG2 were undetectable in either sorted SP or NSP cells (Fig. 3).

### Sensitivity of Sorted 769P SP and NSP Cells to Radiation and Drugs

**Figure 4:**
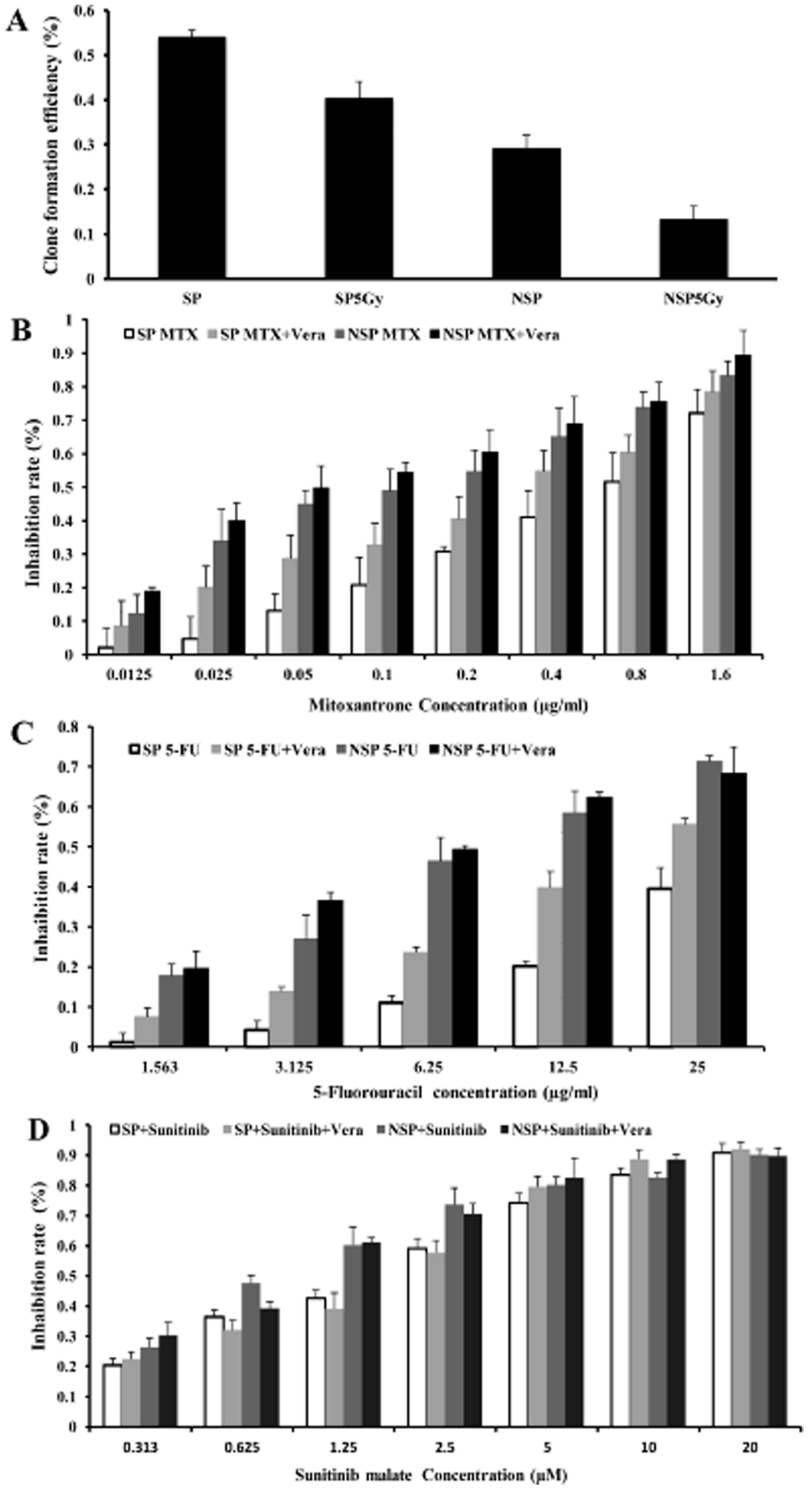
Sensitivity of sorted 769P SP and NSP cells to radiation and chemotherapeutic drugs. **A**, the clone formation efficiency of sorted 769P SP cells is significantly higher than that of NSP cells either before or after 5 Gy of X-ray irradiation. The clone formation efficiency of SP cells is significantly higher than that of NSP cells after irradiation (*P*<0.01); the clone formation efficiency of unirradiated NSP cells is significantly higher than that of irradiated NSP cells (*P*<0.05). **B**, newly sorted 769P SP cells are more resistant to mitoxantrone than NSP cells (*P*<0.01), whereas this resistance is reversed with verapamil pretreatment. **C**, SP cells are also more resistant to 5-fluorouracil than NSP cells (*P*<0.05). The resistance could also be reversed with verapamil pretreatment. **D**, the sensitivity of SP cells to sunitinib is similar to that of NSP cells (*P*>0.05).

We measured the sensitivity of sorted 769P SP and NSP cells to radiation by clone formation assay. The clone formation efficiency of SP cells was significantly higher than that of NSP cells either before or after irradiation (*P*<0.05; Fig. 4A). In detail, the clone formation efficiency of SP cells did not change remarkably after radiation (*P*>0.05), whereas that of NSP cells decreased dramatically (*P*<0.05), suggesting that SP cells were more resistant to X-ray damage than NSP cells.

We also measured the sensitivity of sorted 769P SP and NSP cells to MTX, 5-FU, and sunitinib. SP cells showed strong resistance to MTX, whereas NSP cells were sensitive to MTX (*P*<0.001). Verapamil, an ABC transporter inhibitor, enhanced the inhibitory effect of MIX on SP cells, with a proliferation inhibition rate similar to that of NSP cells under the same conditions (*P*>0.05); however, verapamil failed to enhance the effect of MIX on NSP cells (*P*>0.05) (Fig. 4B). We also found that SP cells were much more resistant to 5-FU than NSP cells (*P*<0.05; Fig. 4C), but their sensitivities to sunitinib were similar (*P*>0.05; Fig. 4D).

### Tumor Formation of Sorted 769P SP and NSP Cells in NOD/SCID Mice

**Figure 5:**
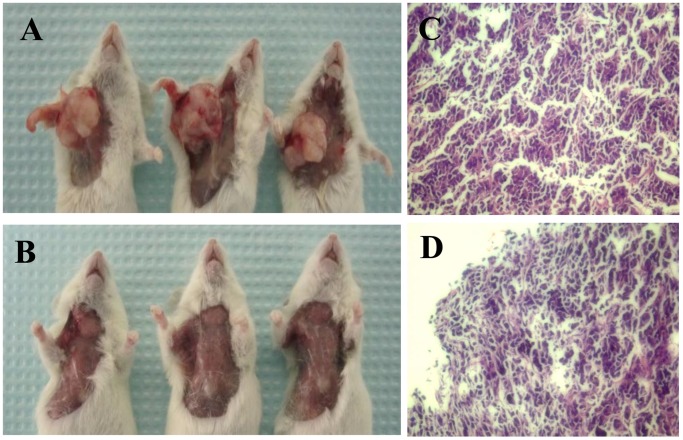
Tumor formation of sorted 769P SP and NSP cells in NOD/SCID mice and pathological examination with H&E staining. A and B, injection sites of NOD/SCID mice at 6 weeks after inoculation with freshly sorted SP or NSP cells. Tumors are observed in all mice inoculated with 2,000 SP cells, but not in mice inoculated with 2,000 NSP cells. C and D, Pathological examination shows typical renal cell carcinoma in mice inoculated with either 2,000 SP cells or 200,000 NSP cells.

Sorted 769P SP and NSP cells were inoculated into NOD/SCID mice to observe their ability to form tumors. One mouse that was inoculated with 20,000 NSP cells and 1 with 200,000 NSP cells died after inoculation. At 6 weeks after cell inoculation, 2 mice developed tumors with only 200 SP cells, whereas the lowest amount of NSP cells to form tumors was 20,000 cells (Fig. 5A; Table 2). Pathological examination confirmed that the tumors formed with SP and NSP cells showed the same characteristics as typical RCC cells just like unsorted 769P cells (Fig. 5B).

**Table 2:** The tumor formation ability of sorted 769P side population (SP) and non-side population (NSP) cells in NOD/SCID mice.

Cell type	The number of mice inoculated with different amount of cells			
	200 cells	2,000 cells	20,000 cells	200,000 cells
SP	2/9	9/9	9/9	N/A
NSP	N/A	0/9	7/8*	8/8*

N/A, not applied. All data are presented as the number of mice developed tumors/the number of mice underwent cell inoculation.

* One mouse died after inoculation.

**Figure 6:**
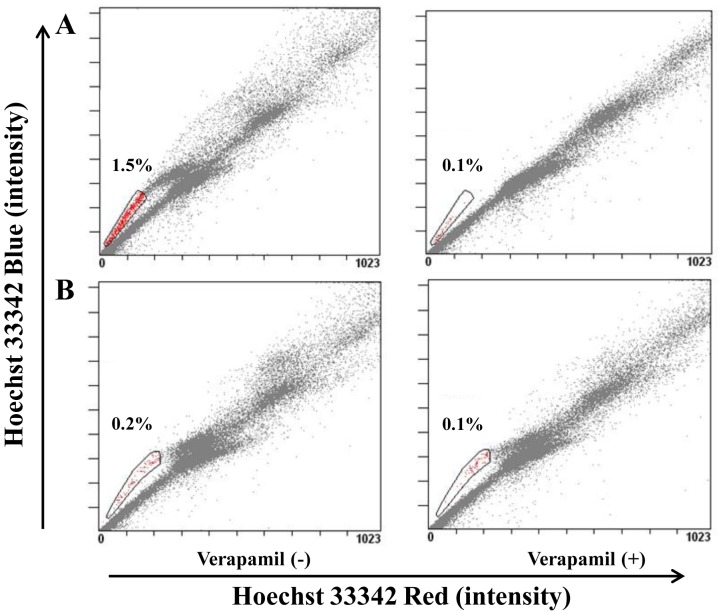
The proportion of SP cells in tumors formed with sorted 769P SP and NSP cells. **A,** cells were dissociated from the tumor formed with 200 769P SP cells; **B**, cells were dissociated from the tumor formed with 20,000 769P NSP cells. The proportion of SP cells was higher in SP cell-formed tumor than in NSP cell-formed tumor (1.5% vs. 0.2%).

To confirm the postulated role of SP cells in tumor formation, a tumor formed with 200 SP cells and a tumor formed with 20,000 NSP cells were dissociated into cell suspension respectively and were stained with Hoechst 33342. FCM analysis showed that the proportion of SP cells was higher in SP cell-formed tumor than in NSP cell-formed tumor (Fig. 6), suggesting the in vivo self-renewal and NSP-differentiation of SP cells.

**Figure 7:**
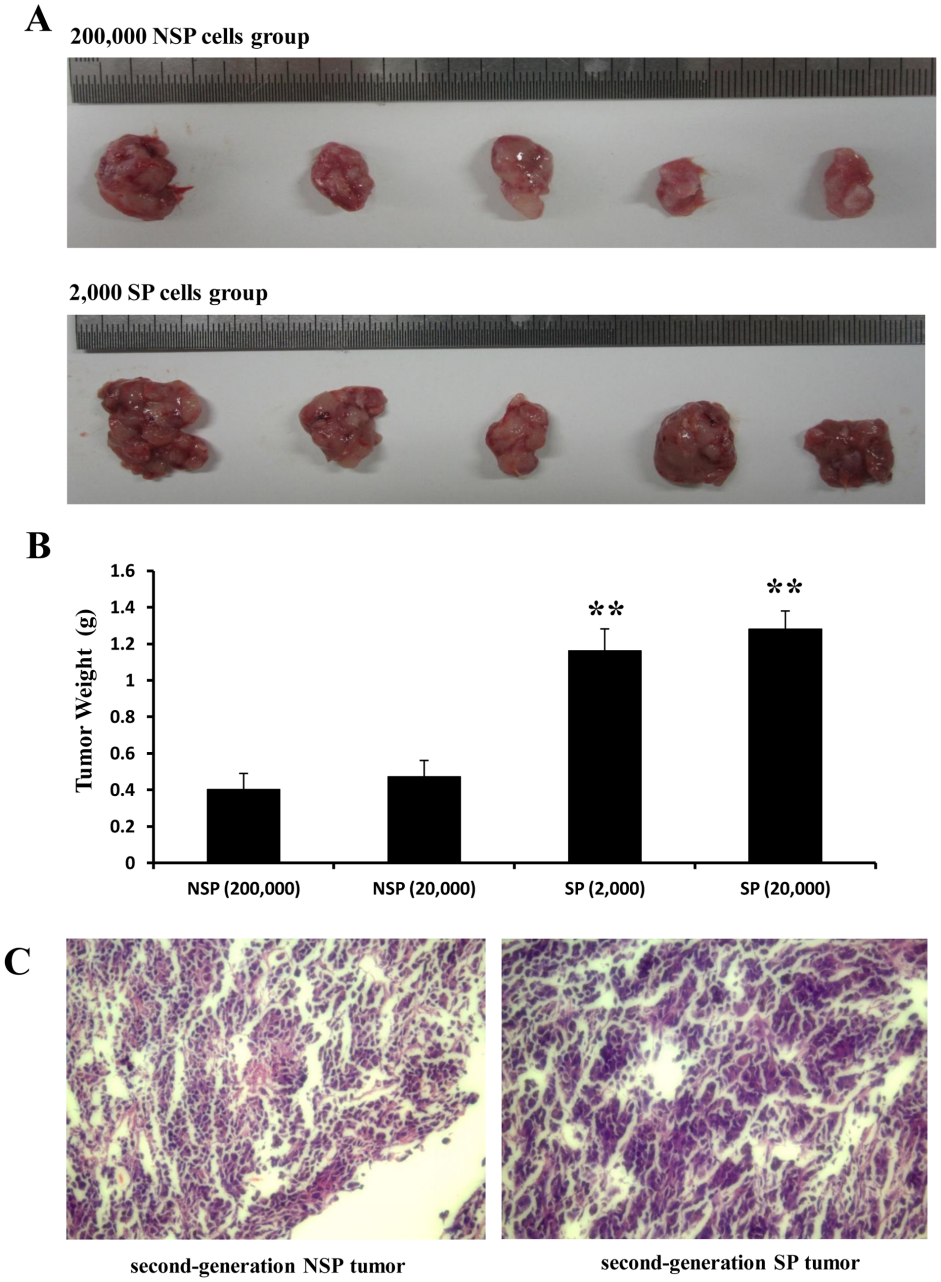
Tumor formation ability of second-generation SP cells in NOD/SCID mice and pathological examination with H&E staining. A, NOD/SCID mice were inoculated with 5,000 second-generation SP cells from a tumor formed with 2,000 SP cells or with 5,000 second-generation NSP cells from a tumor formed with 200,000 NSP cells. Tumors were removed from the mice 6 weeks after inoculation. B, the second-generation SP tumors are significantly heavier than second-generation NSP tumors (** *P*<0.01). C, Pathological examination shows that both the second-generation SP tumor and the second-generation NSP tumor are histologically identical to primary xenograft tumors.

To further determine the tumor formation ability of second-generation SP cells, we inoculated 5,000 SP cells (from 2 tumors formed with 20,000 and 2,000 SP cells, respectively) or 5,000 NSP cells (from 2 tumors formed with 200,000 and 20,000 NSP cells, respectively) into NOD/SCID mice. All mice developed tumors 6 weeks after inoculation. The second-generation NSP tumors were significantly smaller and lighter than the second-generation SP tumors (*P*<0.01; Fig. 7A and B). All second-generation tumors were histologically identical to primary xenograft tumors (Fig. 7C). These results indicated that SP cells were capable of generating tumors serially.

## Discussion

In recent years, researches on CSCs in solid tumors have shown remarkable findings. In this study, we isolated SP cells from human clear cell RCC cell lines to determine biological properties of this cell population.

We found that 4.8% of RCC 769P cells were SP cells, which showed the ability of self-renewal and multi-lineage differentiation. A clone formation efficiency of SP cells higher than that of NSP cells was observed. In addition, SP cells showed tumor formation ability at least 100 times higher than that of NSP cells. Tumors were formed in NOD/SCID mice which were inoculated with only 200 freshly sorted SP cells, whereas at least 20,000 NSP cells were required to form tumors in mice. These results provide direct evidence for the high tumorigenicity of SP cells.

Self-renewal and multi-lineage differentiation capacities are hallmarks of stem cells. Serial transplantation of cells in animal models, although imperfect, can help to evaluate the stability of SP cells in biological behaviors. Our SP re-sorting analysis after serial transplantation of SP cells in mice showed that second-generation SP cells derived from SP cell xenograft tumors maintained the ability of self-renewal and multi-lineage differentiation. Furthermore, cells from both SP and NSP xenograft tumors maintained the capacity to form tumors in mice, but second-generation SP tumors were significantly heavier than second-generation NSP tumors, suggesting that 769P SP cells are more tumorigenic than NSP cells.

Cancer stem cell is considered able to undergo an asymmetrical self-renewing cell division, dividing into one stem cell and one progenitor cell, which could generate a variety of more differentiated functional cells that comprise of the whole tumor society [35]. In our study, the purity of sorted 769P SP cells was 96.61% and that of sorted NSP cells was 99.89%. It is possible that few SP cells may have attached to NSP cells and therefore been collected together. After 10-day culture, the sorted SP cells developed into a community containing 19.51% SP cells and about 80% NSP cells, whereas the sorted NSP cells developed into a community containing only 2.39% SP cells that may arise from few sneaking-in SP cells during sorting. NSP cells can form a few smaller colonies and also regenerated tumors although the tumors were smaller. Taken the findings from long-term differentiation and serial transplantation experiments together, it strongly suggested that SP cells undergo asymmetrical division and are capable of differentiating into NSP cells and forming the bulk of tumor. The capacities of NSP cells to form colonies and tumors, which were much weaker than the capacities of SP cells, may be explained by the small proportion of sneaking-in SP cells among sorted NSP cells.

The SP phenotype is determined by the statuses of ABC transporters ABCB1, ABCC1-5, and ABCG2 [36]. Thus, SP cells are sorted through measuring the rapid efflux of lipophilic fluorescent dyes by ABC transporters [16]. Most previous studies have focused on the function of ABCG2, while the necessity of the pump function of ABCB1 in stem cell expansion was under debate [37]. In our study, the expression of ABCB1 was high in SP cells but low in NSP cells as detected by both RT-PCR and Western blotting. Interestingly, neither ABCC1 nor ABCG2 was expressed in SP and NSP cells, suggesting that only ABCB1 contributes to the function of SP phenotype in 769P cells.

According to the CSC theory, CSCs in solid tumors are resistant to chemotherapy and radiotherapy, and resident CSCs that survived treatment may reform tumors [38]. We conducted chemosensitivity and radiosensitivity assays to compare the sensitivity of SP and NSP cells to conventional therapies. We found that SP cells were more resistant to radiation than NSP cells. In addition, SP cells were more resistant to MIX and 5-FU than NSP cells, indicating that SP cells are widely resistant to conventional chemotherapeutic drugs. However, this drug resistance could be reversed by pretreatment with verapamil, an ABC transporter inhibitor, suggesting that ABC transporters may be responsible for drug resistance [36], [37], [39].

In conclusion, our study proved that SP cells isolated from the RCC 769P cell line possess stem cell characteristics through both *in vitro* and *in vivo* experiments. These SP cells are characterized by strong proliferation potential, self-renewal, differentiation, resistance to chemotherapy and radiation, and *in vivo* tumor formation ability. ABCB1 has been found to contribute to the function of SP cells. Hopefully, developing a therapy targeting this cell population will help to improve the prognosis of RCC.

## Supporting Information

Figure S1 **SP cell sorting results in human renal cell cancer cell lines 786-O, OS-RC-2, SN12C, and SKRC39 using Hoechst 33342.** Only a few SP cells were detected among 786-O and OS-RC-2 cells, and the percentage of SP cells dropped when the cells were pre-incubated with verapamil for about 30 min. No SP cells were detected among SN12C and SKRC39 cells.
(TIF)
