# Supplementary figures and images for: Cancer Stem Cell-Like Side Population Cells in Clear Cell Renal Cell Carcinoma Cell Line 769P
Source: PLoS One. 2013 Jul 11;8(7):e68293. doi: 10.1371/journal.pone.0068293 (PMC3708929; doi:10.1371/journal.pone.0068293)

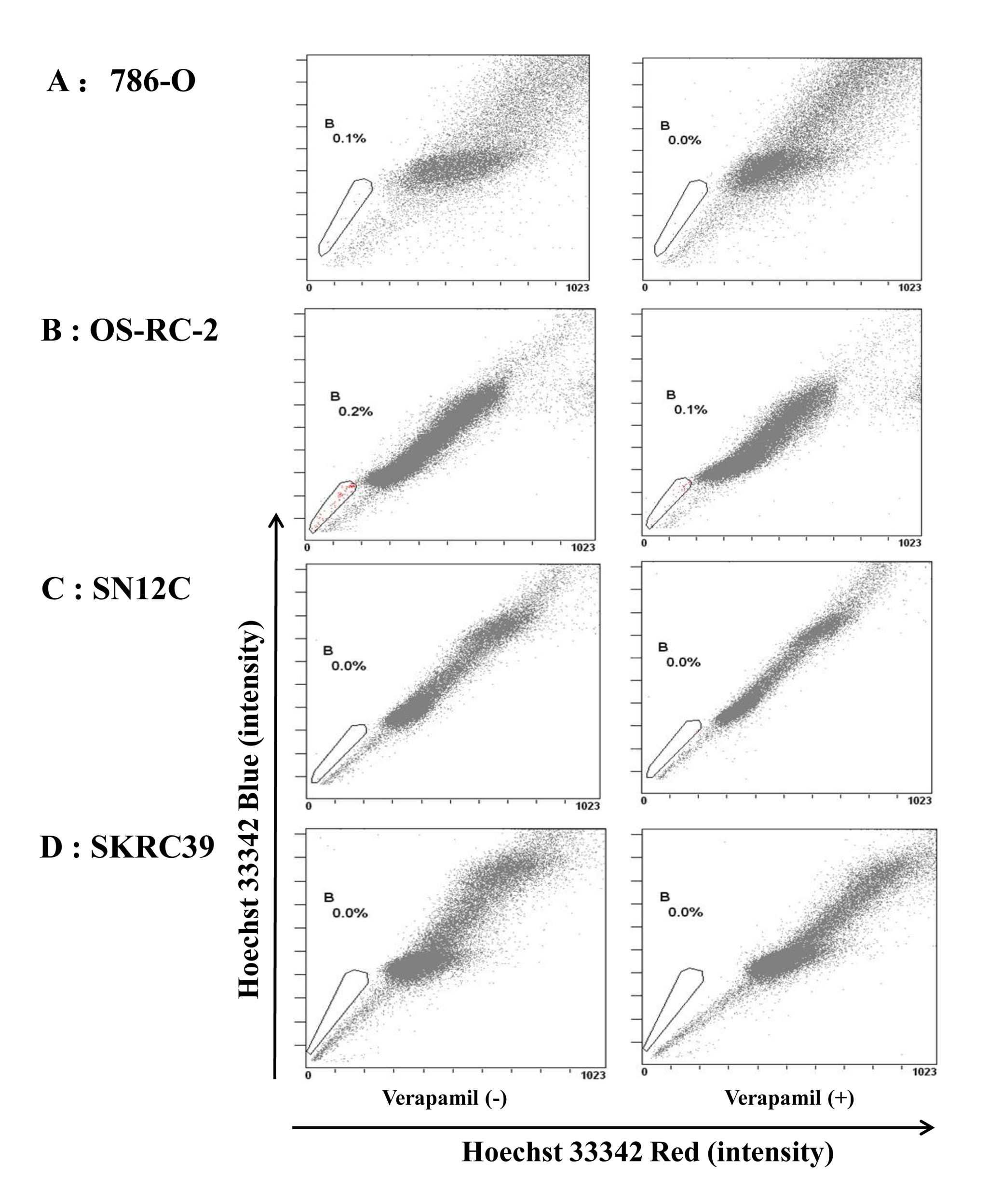

Supplement: Figure S1 — SP cell sorting results in human renal cell cancer cell lines 786-O, OS-RC-2, SN12C, and SKRC39 using Hoechst 33342. Only a few SP cells were detected among 786-O and OS-RC-2 cells, and the percentage of SP cells dropped when the cells were pre-incubated with verapamil for about 30 min. No SP cells were detected among SN12C and SKRC39 cells. (TIF) [file pone.0068293.s001.tif]
